# Development of a Methodology Using Artificial Neural Network in the Detection and Diagnosis of Faults for Pneumatic Control Valves

**DOI:** 10.3390/s21030853

**Published:** 2021-01-27

**Authors:** Ana Andrade, Kennedy Lopes, Bernardo Lima, André Maitelli

**Affiliations:** Laboratory of Petroleum Automation—LAUT, Federal University of Rio Grande do Norte-UFRN, Natal 59078-970, Brazil; acca.engpet@gmail.com (A.A.); bernardo1411@hotmail.com (B.L.); maitelli@dca.ufrn.br (A.M.)

**Keywords:** fault detection and diagnosis, artificial neural network, NARX, control valve, decision tree, signature matrix

## Abstract

To satisfy the market, competition in the industrial sector aims for productivity and safety in industrial plant control systems. The appearance of a fault can compromise the system’s proper functioning process. Therefore, Fault Detection and Diagnosis (FDD) methods contribute to avoiding any undesired events, as there are techniques and methods that study the detection, isolation, identification and, consequently, fault diagnosis. In this work, a new methodology that uses faults emulation to obtain parameters similar to the Development and Application of Methods for Diagnosis of Actuators in Industrial Control Systems (DAMADICS) benchmark model will be developed. This methodology uses previous information from tests on sensors with and without faults to detect and classify the situation of the plant and, in the presence of faults, perform the diagnosis through a process of elimination in a hierarchical manner. In this way, the definition of residue signature is used as well as the creation of a decision tree. The whole process is carried out incorporating FDD techniques, through the Non-Linear Auto-Regressive Neural Network Model With Exogenous Inputs (NARX), in the diagnosis of the behavioral prediction of the signals to generate the residual values. Then, it is applied to the construction of the decision tree based on the most significant residue of a certain signal, enabling the process of acquisition and formation of the signature matrix. With the procedures in this article, it is possible to demonstrate a practical and systematic method of how to emulate faults for control valves and the possibility of carrying out an analysis of the data to acquire signatures of the fault behavior. Finally, simulations resulting from the most sensitized variables for the production of residuals that is generated by neural networks are presented, which are used to obtain signatures and isolate the flaws. The process proves to be efficient in computational time and makes it easy to present a fault diagnosis strategy that can be reproduced in other processes.

## 1. Introduction

Currently, with industries presenting an increasingly competitive profile, companies in the oil, petrochemical and natural gas sector, among others, are beginning to demonstrate a need for improvement in productivity, reliability in system stabilization and safety of their industrial plants.

Although there is a continuous search for increasingly efficient productivity, safely and with quality, the search for improvement in processes can be threatened due to the need for the accelerated use of the equipment that make up an industrial plant, making them increasingly likely to show signs of degradation such as: wear due to incorrect operation or repetitive movement, the principle of corrosion, the appearance of erosion, the accumulation of debris (sedimentation), the discovery of an (obstruction), among others. The appearance of these symptoms can be an indication of the presence of abnormalities in the system and qualify failures that can lead to a permanent interruption in the ability to perform a certain function during operation [1].

Although the controllers are able to meet several types of disturbances, there are some changes, during the process, that they are unable to deal with correctly. These unexpected situations can characterize a fault, which can be defined as being an unallowed deviation from at least a certain characteristic property or process variable of the system [2,3].

In this respect, faults in the process, production and power generation industries, among others, can be classified according to their type as faults of actuators, sensors and plant components or parameters. These possible failures interrupt the action in the control process and they can produce substantial measurement errors or even change the dynamic input/output properties of the system, causing an increase in operating costs and even a degradation in the performance of the industrial plant [4,5,6].

In the industrial processes, to solve problems, early diagnosis can allow the performance of necessary procedures such as prevention. Thus, fault detection becomes a fundamental measure in the system control process, consequently, the importance of developing reliable methodologies for the diagnosis of the system becomes essential [7]. Therefore, FDD techniques can be used to monitor the functioning of systems, detect anomalies and in the occurrence of faults, be able to classify them. The FDD has been acting in research for many decades with different applications, aiming in the most diverse areas to solve problems through the control of industrial processes which, as presented in [8], the search for well-designed controls results in increased system reliability, to avoid the occurrence of faults. Other applications of FDD techniques can be related to the use of technologies in building constructions as seen in [9], whose work mentions a situation contrary to the physical redundancy process, in which virtual redundancy does not increase the cost and complexity, thus bringing, benefits such as the application of automatic FDD methods which, in turn, allows the comparison of duplicate signals, making it possible to detect divergences from each other. Then, fault-tolerant control can be achieved by duplicating a physical sensor with a virtual one so that the system can continue to function even if it fails. Still, with a wide range of applications, a review study is presented in [10], based on automated methods of detecting and diagnosing faults in heating, ventilation and air conditioning systems [11]. As previously introduced, we know the importance of a stabilized control loop. Then, in [12] the author proposed a method capable of detecting faults in the pneumatic control valve to obtain better mesh stability through a technique that determines the fault in a valve through the analysis of the output vibration data of the valve. We can also include the use of FDD techniques in the approach of neural networks, in [13] in the process of detecting and isolating faults in the control valve, a conventional detector that aimed to correct errors in the fault threshold regions, received as complement a neural network, whose proposed detector that included the network design, resulted in a better performance in the faults detection and isolation stage.

In the field of engineering, a certain relevance was observed in articles that use neural networks to solve Fault Detection and Isolation (FDI) and FDD problems, although the development of research with neural network applications is not something so recent [14,15,16,17,18]. Its use in fault detection has been extensively studied, showing wide applicability for nonlinear systems in the context of recurrent networks, which is why researchers explore its use with purposes for several general predictive finalities, especially when referring to process control [19,20,21,22,23]. Thus, several scholars have noticed neural networks as something promising that would represent the knowledge of the faults and their classification [24,25,26,27,28]. Therefore, as a trend in the faults classification stages, data fusion has been one of the main areas of focus in data analysis, used by some authors for the development of research. Among the several works that approach data fusion as a proposed methodology, some have as main objective the use of a data fusion of multiple sensors applied to the method of detection and classification of faults in distributed physical processes for onboard auxiliary systems, resulting in the generation of heterogeneous data by the dynamics of the interactive process [29]. With the same principle in the use of the data fusion technique, this other work proposes an adaptive multisensor fusion method based on Deep Convolutional Neural Networks (DCNN) for fault diagnosis, the results reported better diagnostic accuracy in relation to the comparative methods in the experiment [30]. In [31], based on detailed data fusion research, a Hybrid Deep Belief Network (HDBN) learning model was developed, capable of integrating data in various ways for the intelligent diagnosis of faults applied to a vehicle drive system. Then, as a proposal, three data fusion methods were developed: data union, data join and data hybrid, in order to improve the overall performance of the proposed model without collecting more data.

Finally, in [32] the author presented a method of fault diagnosis based on Improved Detrended Fluctuation Analysis (IDFA) and multisensor data fusion, with applicability for rolling element bearings, proving the effectiveness of the proposed method through the validation of experimental data.

With an overview of the works already mentioned related to data fusion, it was possible to verify that when combining and analyzing measurements together, an appropriate approach to detect failures of complex systems was observed specifically in order to obtain safer and more efficient processes.

As an example of application of the presented methodology, an initial study of the faults that are presented in the DAMADICS benchmark was used to evaluate different types of faults through tests that do not necessarily need to have defective valves. The procedure is carried out through the emulation of signals, which intend to incorporate in a controlled system, situations that could occur in the wear of the equipment. The signals resulting from these emulations determined a database used in the selection of the most sensitized variables for the production of residual patterns generated by neural networks. Once the plant signals of faults situations are captured, neural networks are trained to learn how the output behaves. Thus obtaining different neural networks trained and expert for each behavior. Then, techniques of the neural network NARX were used to predict the behavior of the signals. The residues are generated by comparing the signal predicted by the NARX network with the real values of the plant, in order to present this information for the FDD methods.

We decided to use this methodology to make the system for detection and diagnosis as autonomous as possible. The main objective was to define a way to analyze the faults without having to adjust several parameters. When we work with artificial neural networks we have the advantage of offering generalizations to the problems that, once trained and understood by the networks, the settings are regulated directly by the synaptic weights and not at the discretion of the system specialists.

For the process of applying the FDD methods, a combination models method is performed. The data-oriented model with the use of the neural network for the generation of residues, fundamental for the faults diagnosis stage, and the other model is based on rules in the patterns classification, that is, the specialist’s knowledge, in the case of network, which must be developed to determine a set of rules describing the behavior of the system. Thus, some faults will be described by rules, allowing better identification and precision in the diagnosis. Then, for the FDD process, the decision tree will be built to assist in the diagnosis of the most significant residue for a given signal. A decision is made at each new level of the tree by signing the standards that provide the fault isolation.

The article presents as novelty the application in an iterative way in the search of the faults diagnosis. The procedure is based on tracing the faults through a decision tree that eliminates the possible causes of the faults. In this sense, an innovative method of building this decision tree was introduced, composed of a set of neural networks to detect and isolate the flaws learned from the signals tests. The rest of the article is organized as follows: in Section 2, the proposed method is described in detail. Section 3 presents the case study, Section 4 results and analysis and finally, the conclusions are given in Section 5.

## 2. Materials and Methods

In this section, we describe the development of the methodology for the detection and diagnosis of failures using neural network techniques.

As a necessary step to substantiate the work, the plant must maintain the same conditions for each type of fault, such as level stability, flow maintenance, pressure control, among others. As a result, the variables characterized as defective may sensitize only some signs of interest. In this way, the signals of interest will be defined as those with the greatest differential of means and variances for each specific fault. The most suitable situation for identifying the behavior of a fault is characterized by the ability that each fault has to produce different behavior in the plant. Thus, an intelligent system can learn to diagnose the fault.

**Figure 1:**
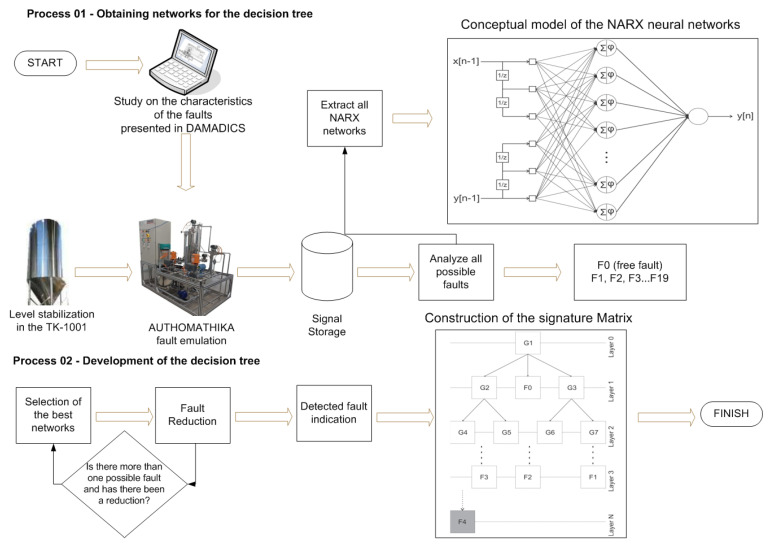
General flowchart of the methodology developed, applied to the study of faults for pneumatic control valve.

In order to make the understanding of the FDD method more evident, the high-level architecture can be separated into two stages, as described below according to the flowchart of Figure 1.

At first, a study is carried out on the effect of each fault with certain intensities. This preparation is carried out based on the work on DAMADICS and adapting the situations of faults in the emulation of AUTHOMATHIKA. With that in mind, the controllers are adjusted to try to stabilize the tank level (TK-1001) even if faults are occurring. The robustness of the controller guarantees an adequate level for the plant, but for this reason, some sensors perceive an unusual activity when compared to normal situations. These perceptions are affected by the intensity of the fault and its characteristics. Then, all relevant signals from the plant are captured and stored for future analysis. The analyses are performed according to which will be the best neural network capable of producing the best residue (with the least approximation error). This residue is defined as the difference in the time signals from the neural network, which simulates the plant’s behavior, with its value stored in times of fault.

After analyzing and extracting the best networks for each fault situation, the development of the decision tree is put into action through the fault isolation procedure. This second process develops the elimination of faults until the moment that the candidates can no longer be eliminated, thus finalizing the diagnosis. The conclusion will not always be unique and the results will have to be analyzed by specialists for a refinement, whose wear is causing this behavior. However, in this methodology, we present an approach on how to reduce the investigation of possible causes of the present behavior.

As seen in Figure 1, the necessary condition to start the whole process was the control of the already static level, which allows the simulation of all faults, from F0 that characterizes the situation without the occurrence of the fault until Fault F19. Thus, the data were acquired and analyzed for the selection of certain variables, such as, for example, inlet and outlet flow rate, actual valve opening, desired valve opening, among others. In this way, assisting the diagnosis, it was possible to analyze all the faults for the process of the next step referring to the construction of the decision tree for the generation of residue, which was analyzed according to the performance of the faults for the construction of the matrix of signatures, based on the evaluation of the residual signal.

**Figure 2:**
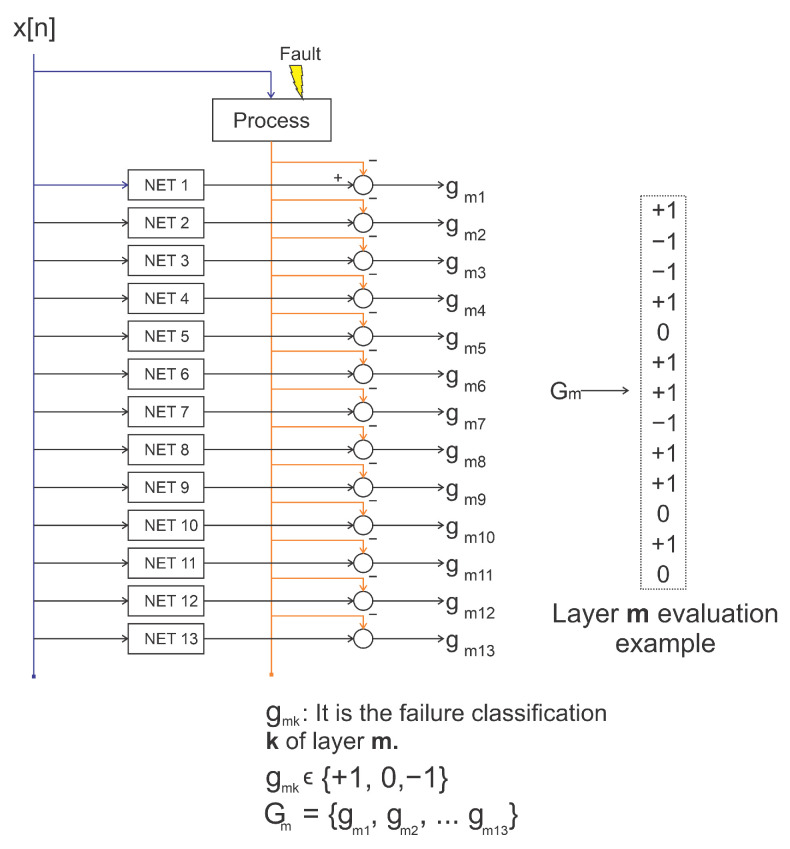
Obtaining the fault signature by analyzing the thresholds present in the residues.

The signature matrix was developed according to Figure 2, whose trained neural network must be chosen specifically to determine which network best classified a given fault.

As shown in Figure 2, the signature matrix was developed to relate each neural network as a specialist in observing each type and intensity of fault. For example, a given fault has the most appropriate diagnosis according to the network, which will keep the residue within the defined thresholds. Situations like these demonstrate that the residue from this NARX gets an indication of zero in the signature. Values of +1 or −1 occur when the residue is above or below these limits, respectively.

Networks are trained to become experts in detecting each type of fault. In the previous analysis of the sensors, different network configurations are trained with all fault situations that may occur in the plant. At the end of this procedure, the neural networks are organized in order of the best and worst network in the classification for each type of failure. In the example shown in Figure 2, 13 neural networks specialized in detecting 13 plant situations are presented: the first indicates the possibility of nonoccurrence of the faults and the rest showing that the faults of (F2 … F13) may be active.

The presentation of a signature represents a step in the construction of the decision tree. Diagnostics are performed at classification levels by re-evaluating the data. The data will be evaluated until the result indicates only an active neural network or that the active neural networks can no longer be separated to distinguish the fault. In this way, a new structure similar to Figure 2 is used with the same data presented in the previous layer but using the second best diagnostic networks according to the categorization developed in the training process. The process continues until there are no more discarded networks, which are the networks in which the activations obtain a response of 0.

From the interest in the detection and diagnosis of faults, neural network techniques can be applied; in this case, the NARX was used as a viable tool in the training process for analysis in the detection of faults in order to guarantee their time series forecasting effectiveness and consistency.

The NARX networks were chosen because they can incorporate the dynamics of process signals. To make this possible, it is important to use exogenous inputs that are the feedback of your outputs. In this sense, networks were trained to incorporate the dynamics of the signal residues: the difference between the signal presented and an approximation of the specialist NARX network for each fault.

**Figure 3:**
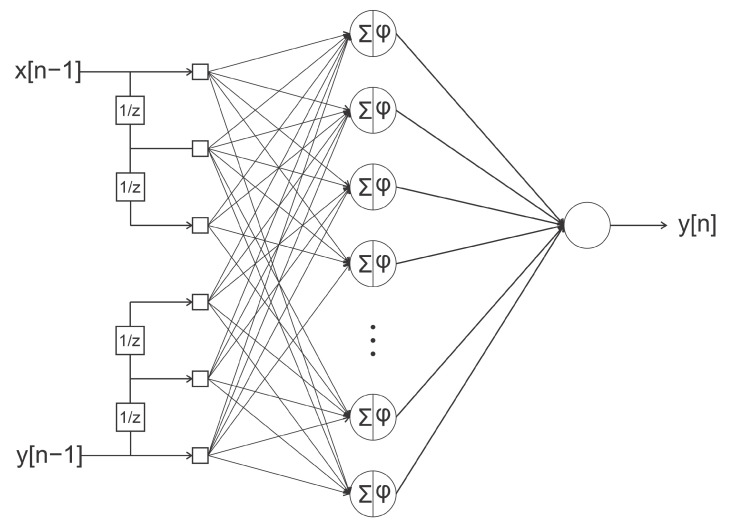
Conceptual model of the NARX neural network.

Thus, different configurations of NARX neural networks were designed, shown in Figure 3. As the training data were available, two types of network structures were tested, one in which the inputs y[n−1] are fed back by the output itself to produce the y[n] and another input x[n−1] where the output is produced with the values y[n] approximated by the values made available offline of the emulations of the faults.

The proposal to use the NARX neural network is defined to simulate the behavior of the faults from the perspective of each one. With this, when the process is being carried out, the neural network, together with the actual outputs of the plant, will cause the residue necessary for the definition of the signatures.

As part of the proposed methodology, a code was developed that makes it possible to systematically train neural networks for different elaborated structures. Tests and analyses were applied to compare their performances, so that the choice of the best neural network topology to be used for each specific fault with certain predicted intensities, presents some relevant aspects: delays in the feedback output, different amounts of neurons in the single hidden layer and definition of the input-output pattern. Thus, the stage of searching always for the best network in relation to a certain condition allows for the presentation of the following structure:

- Delays at the exit;
- With or without feedback;
- Single hidden layer with varying amounts of neurons;
- Definition of the input-output standard.

For training with all standards that have or do not have faults, applied to each number of neural networks, the best networks should be presented as results for the training, validation and test phases.

In order to ensure that the results of the neural networks are consistent with the defined topology, it is recommended that the training procedure be repeated a few times. The methodology idealized in the search for the best network will be finalized when the fault detection occurs efficiently, deciding for only one fault or when the system is no longer able to distinguish between possible faults.

In the presence of a chain of the best networks to determine each situation, groupings with different patterns can be created that best suit a given network belonging to the first layer. Thus, Artificial Neural Network (ANN) that is not suitable for that circumstance will then be discarded. This process is repeated with the other auxiliary layers using tiebreakers until the moment the fault is isolated.

The best topologies are those whose signal simulation with the neural network has managed to keep the output within the pre-established limits of the appropriate situation, that is, when it is previously established which are the best neural networks, which implies that they are the networks that manage to keep the signal forecast within the limits of what would happen if a fault of that presented pattern occurred.

For the construction of the decision tree in the residual assessment process, the detection step will be essential for the analysis of the magnitude of the residues. In a first analysis, not all residue will have a zero value, nor will a single network indicate this value. However, this pattern composed of all networks, previously known through training, indicates what is happening in the plant.

**Figure 4:**
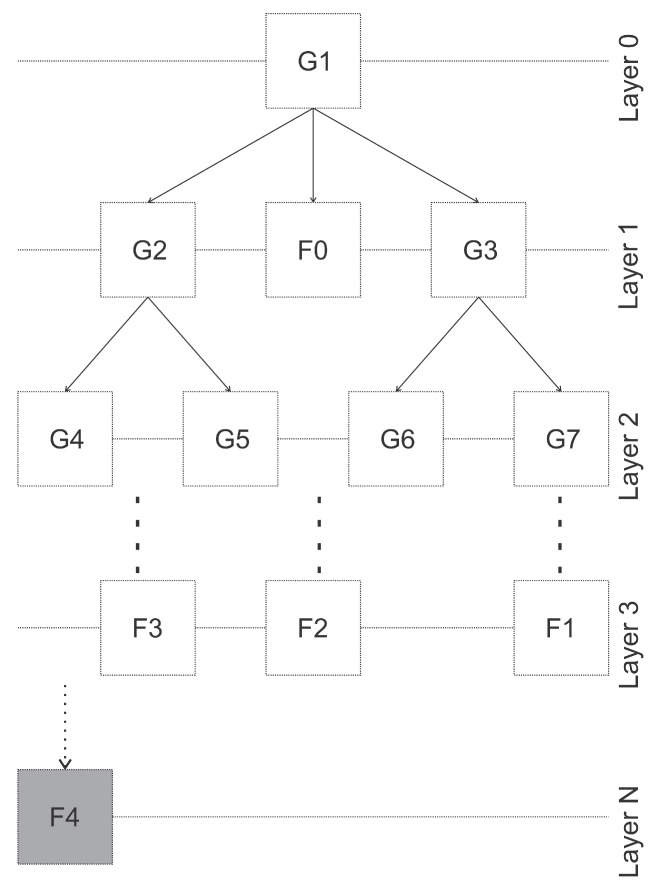
Logical representation of fault signature layers.

Even so, there may be doubts about what is happening. As all networks are being analyzed simultaneously, other situations for diagnosis are evaluated. This procedure is presented by an analysis scheme of other layers of clusters. This is like the scheme shown in Figure 4.

We trained 48 neural networks and classified them as to their performance in diagnosing each of the 12 faults as well as the normal situation. Thus, there is a priority on what are the best settings for each type of pattern. If there is any doubt that the network has not adequately isolated a single type, these hypotheses will be analyzed by another layer that will examine another group of NARX networks. It is not necessary to present all the data again for analysis, they must immediately go through all networks and only the sets that indicate the groups are analyzed.

The decision tree has a hierarchical structure made up of branches in order to simplify the process. Signatures contribute to the detection of faults, and their isolation is aided by taking advantage of the tree structure.

## 3. Case Study

With the development and progress of science and technology, the research and application of methods using FDD attract more and more attention and, thus, many results are obtained. Thus, different approaches have been used when applying FDD techniques in order to solve problems [33].

An example of these applicabilities is the use of techniques to describe different perspectives related to hybrid methods in the detection, identification, behavior and diagnosis of faults from residue, including in the presence of noise as well as in the process of building decision trees [34].

**Figure 5:**
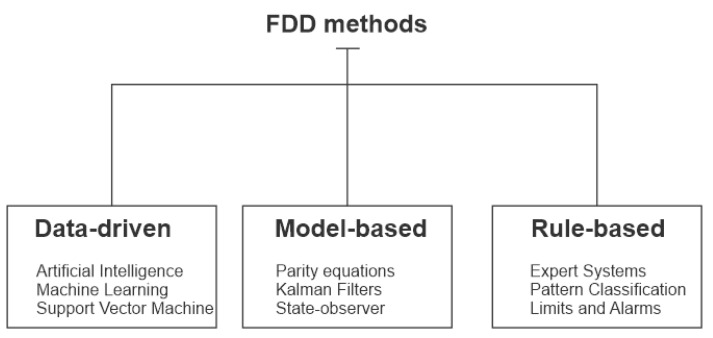
Diagram adapted from the classification of FDD methods [9].

Another categorization regarding its applicability provides a synthesized classification of the FDD methods and is represented in the diagram in Figure 5. However, we can extrapolate the field of applications in other processes and or systems [9].

According to this classification of the FDD methods presented, the present work brought together two of these techniques to obtain a practical procedure for discovering valve faults. While we have an automated data collection process for each fault, using a data-oriented methodology, the classification of these data follows a system of rules characterized by the generation of signatures, more precisely the known patterns of faults that are diagnosed through a vector of characteristics produced as a comparison of the plant with NARX neural networks.

To apply FDD techniques and use ANN for the development of the methodology, it made it possible to make the system autonomous for the detection and diagnosis process, with the main objective being to determine a way to analyze the faults without having to adjust several parameters. Thus, when we work with ANN we have the advantage of offering generalizations to the problems that, once trained and understood by the networks, the settings are regulated directly by the synaptic weights and not at the discretion of the system specialists.

Then, in this section the case study will be presented, which was based on the DAMADICS actuator system, well known in the literature for being a benchmark project, enabling studies related to the fault detection in valves, mainly in the area of engineering in process control. In this work, the set of faults of the DAMADICS benchmark was used as a base model to be emulated and tested in the didactic plant AUTHOMATHIKA PDH-1002, thus making it possible to validate the proposed methodology for the detection and diagnosis of faults using neural networks and, finally, the description of the analysis of the results obtained.

### 3.1. DAMADICS

**Figure 6:**
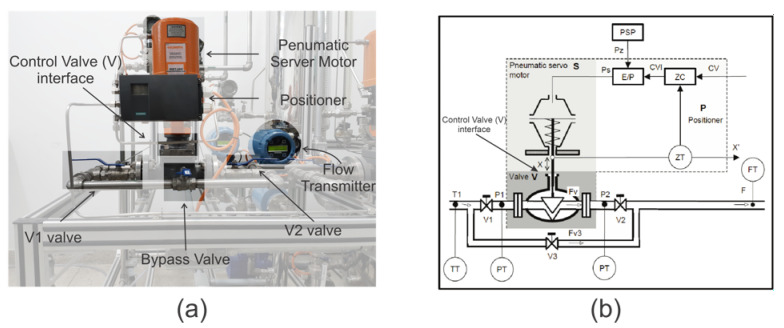
Similarity scheme of actuators: (**a**) AUTHOMATHIKA actuator based on (**b**) actuator model DAMADICS [35].

The DAMADICS simulator integrates the benchmark model that was developed according to descriptions from a real industrial actuator. This simulator aims to control the flow of water from a boiler that is part of an evaporation station. Its physical composition consists essentially of three elements, as seen in Figure 6: the control valve (V), the pneumatic servomotor (S) and the positioner (P). These three main components are basically composed of a set of physical measurement values: flow sensor measurement (F), pressure sensor at the valve inlet (P1), pressure sensor at the valve outlet (P2), liquid temperature (T1), stem displacement (X) and process control external signal (CV). As for the (V1), (V2) and (V3) valves, they can be activated manually in case of any eventuality, thus, the alarm on the actuator must trigger for the closing of the (V1) and (V2) valves, as well as the manual control by the (V3) valve [35,36].

For the case study, the context of the applicability of the fault simulator used DAMADI CS and the didactic plant AUTHOMATHIKA is not related to their differences but to the conceptual use of the faults of DAMADICS, which were emulated in AUTHOMATHIKA. This process resembles the real situation due to the analysis of signals, since AUTHOMATHI KA is a physical plant of laboratory size, for educational purposes, emphasizing the supervision and instrumentation of systems, as well as the process of identification and control of faults.

In the detection and identification of faults, the composition of the benchmark model makes it possible to simulate a set of nineteen faults (F1, F2, … F19) of the actuator, classified into four distinct groups: control valve failures (F1 … F7); pneumatic servomotor faults (F8 … F11), positioner faults (F12 … F14) and general faults/external faults (F15 … F19). Thus, the faults previously chosen by the DAMADICS actuator to act in the didactic plant were: obstruction fault (F1), sedimentation fault (F2), and erosion fault (F3) [37,38].

All of these selected faults belong to the fault class in the control valve and are related to the valve internals. They were studied and analyzed as to their type and mode of action, classified as incipient, occurring gradually or abruptly, faster. However, some problems can still result in failures of the type: grabbing, external and internal leaks present in the control valve, as well as faults in the pneumatic actuators, occurred due to certain complications in the connection or connector, springs and even, diaphragm or piston. The valve model shown in Figure 6 is known as a globe valve. The types of faults on that device are already cataloged and once the fault is discovered, it is possible to correct it. As an example, it may happen that the stem moves with difficulty or even remains stationary. In this situation, the probable cause may be insufficient air supply, the positioner is malfunctioning or even the force of the actuator is insufficient. As a corrective action: check if there is an air leak in the actuator or an instrument signal; the positioner must be consulted in its installation, operation and maintenance manual; check the pressure of the actuator supply. A description of another type of fault that can occur in this valve is excessive leakage through the valve seat. This may be due to incorrect shutter adjustment, the shutter may be worn or damaged or the seat is worn or damaged. As a corrective action: reassemble the valve for the correct adjustment of the shutter; disassemble the valve to replace the shutter; disassemble the valve to change the seat.

### 3.2. AUTHOMATHIKA PDH-1002

**Figure 7:**
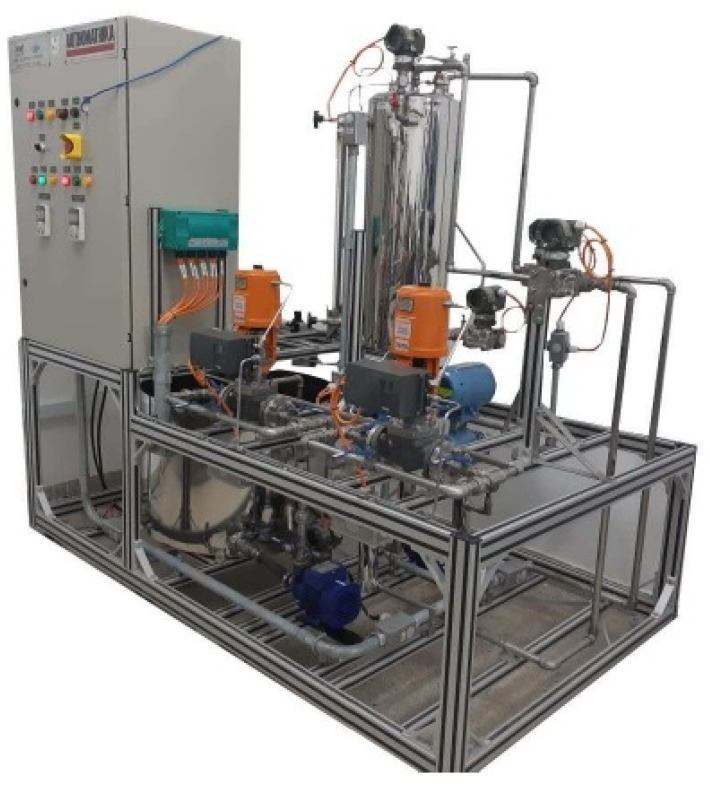
Didactic plant AUTHOMATHIKA PDH-1002.

The AUTHOMATHIKA plant identified by Figure 7 was developed for use in research projects at the Petroleum Automation Laboratory (LAUT-UFRN). The aim is to facilitate teaching in control and identification of industrial systems, offering various resources and operational facilities. The plant also has several predefined and illustrated meshes in the Processview Supervisory System, assisting the control process.

**Figure 8:**
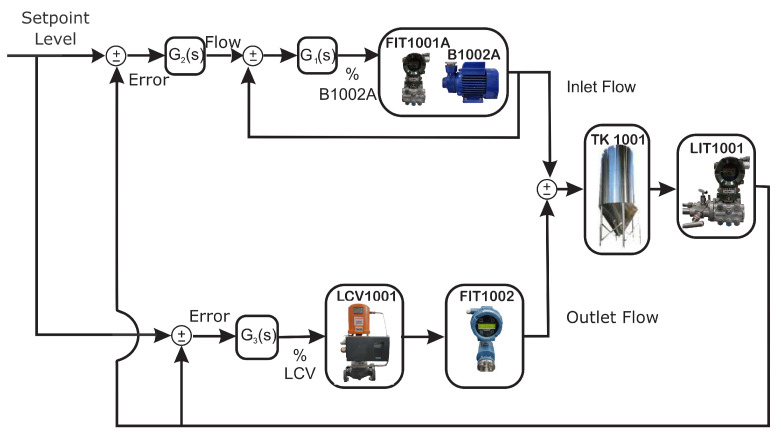
Control grid of the didactic plant.

Analyzing through Figure 8, as an essential step for the development of the work, it was necessary to establish the tank level control (TK-1001) with the setpoint value already set at 30 cm and compare it with the real level value. Through the manipulation of the inlet and outlet flows, the $G_{2}$(s) controller acts on the top of the tank, determining through the difference between the intended level and the desired setpoint what should be the flow required to eliminate the level error. The inlet flow of the tank (desirable) must be compared with the outlet flow (intended) obtained by the plant sensors so that it is presented to the $G_{1}$(s) controller. The same controller uses the difference of the desired and current flow rates, to determine the appropriate frequency to be modified by the frequency inverter itself, in order to keep the pump level (B-1002A) stabilized.

The most external control, on the other hand, allows for the changing of the level through the $G_{3}$(s) controller. This is by means of signals belonging to the levels emitted by the sensors, to the level controller as desired and thus, to describe what will be the intended output flow to manipulate the desired valve opening (LCV-1001) so that the error between the setpoint and the intended level remains close to zero. This whole procedure enabled the didactic plant to be able to work on a static level of the tank, with different failures analyzed under the same conditions. As the outlet flow is dependent on the level and given that the dimensions of the tank are large enough for the outlet flow to be significant, good control was necessary to be robust in order to ensure that the level does not differ from the operating point.

In the process of modeling the faults for the detection and identification steps, it was necessary to acquire the database of the didactic plant to validate the procedure of the dynamic system that operates presenting situations in a normal (without the occurrence of faults) and abnormal (in the faults). As an initialization step, some signals were evaluated for the selection of certain variables, namely: inlet flow (desirable) from the tank; outflow (intended) from the tank; effective (real) opening of the valve; the desired opening of the valve, and finally, the variable level of the tank which must always operate stably due to the control of the flow system level of the didactic plant, thus, there is no possible destabilization that will interfere in the identification of the detected faults.

In the simulation, to present the normal behavior of the valve, what was emitted in the actuation necessarily occurs in the effective opening. As the valve itself has an internal control and consequently, its dynamics between the actuation signal and the one returned by the positioner, the differences may not always constitute an actuation fault. Then, the fault assessment process (F1, F2 and F3), already presented in the DAMADICS section, presented an alternating reference signal that was sent to the valve to cover the entire opening and closing path. If any defect is present, the valve’s behavior should not obey the reference signal but the failed signal. For all faults, the situations were analyzed with different intensity levels: obstruction (10%, 20%, 30% and 40%), sedimentation (90%, 85%, 80% and 75%) and erosion (10%, 15%, 20% and 25%).

The F1 Fault emulation was performed by sending the setpoint signal to the closed 0% level in a stochastic manner. Thus, different intensities of 10%, 20%, 30% and 40% were defined as the probability for the occurrence of valve obstruction at each interval of change of reference signal. In situations where the plant has a simulated obstruction, the tank level tends to increase because the control is not designed for that situation. If the level rises indefinitely during the presence of F1, the defective valve’s behavior cannot be captured in the same situation when the fault ends.

In the F2 Fault, which is characterized by sedimentation, the open valve maintenance time is increased by the controller to compensate for the deficiency of not being able to open completely. The simulation of this fault occurred by limiting the valve travel superiorly, so that the simulation prevents the valve from reaching any value above the determined limit. Something similar happened in Fault F3, in which erosion prevents the valve from reaching a lower value than stipulated.

In the use of the NARX network for the detection and diagnosis of faults, at the stage of the process of training the networks for different structures, the search for the topology of the best network that fits the given fault and specific intensity was developed with the following structure:

- Several exit delay variables, which in this work were used from 1 to 6;
- With or without feedback;
- A hidden layer with different numbers of neurons was applied: (5, 10, 15 and 20);
- Definition of the input-output standard (three inputs for one output).

The proposal to use the NARX neural network is defined to simulate the behavior of the faults from the perspective of each one of them. Thus, when the process is being carried out, the neural network together with the actual outputs of the plant cause the residue necessary for the definition of the signatures.

## 4. Results and Analysis

In the presented methodology, the residues that were within the thresholds were used to filter the faults. The data are evaluated by layers, from neural networks, for the production of residues that are labeled with +1, −1 and 0, representing the logical situations for comparing the behavior of the NARX standard with the process output.

**Figure 9:**
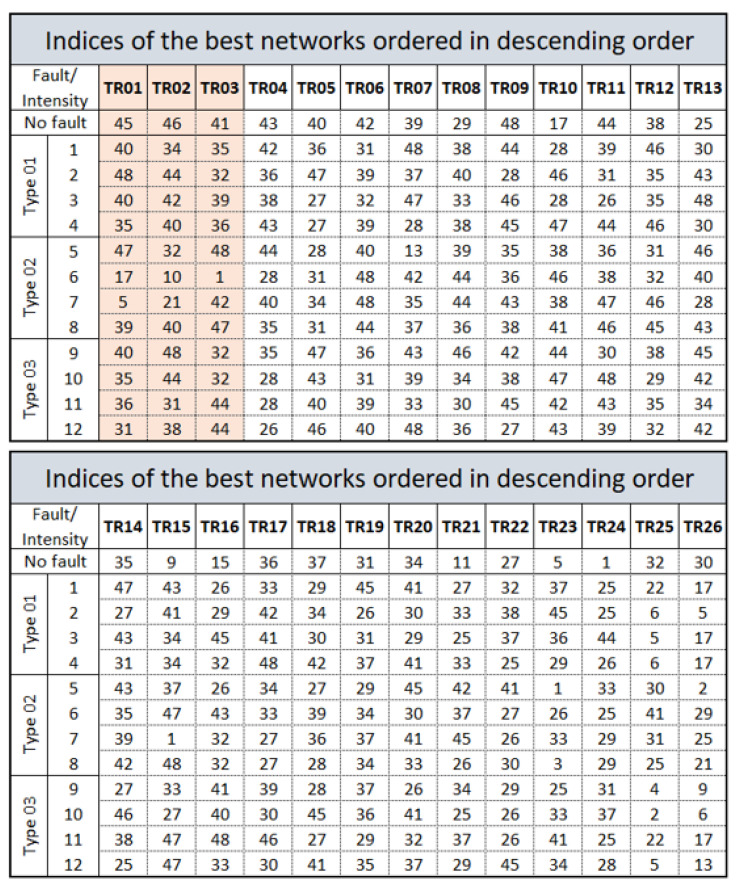
The 26 best networks with the best training performances, in descending order.

The representation of each network can be found by the diagram in Figure 9. The networks are identified by indexes, in which 48 different configurations are defined, trained for all faults and normal situation. As shown in this figure, each neural network was named Training Rule (TR01…TR26), in which the classification of networks specialized in detecting 13 situations present in the plant are exposed.

**Figure 10:**
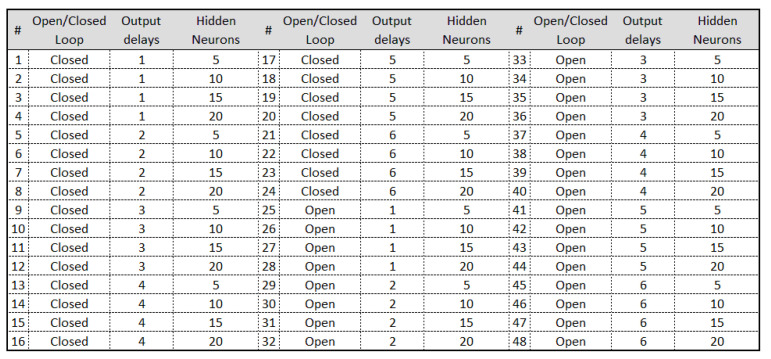
Identification of the networks in Figure 9, in relation to the number of neurons, the hidden layer, delays in the outputs used and whether the network is open or closed loop.

The indexes in Figure 9 must be selected from a set of Cartesian Product, on the chosen properties of the neural network architecture. Each number represents an index that names the neural network itself to detect each type of fault with its due intensity. The existing possibilities and the indexes that identify them are shown in Figure 10. Thus, it is still possible to perform other TR from the total possible configurations of NARX networks.

### Example of a Failed Signal F1—Multiple Network Layers

**Figure 11:**
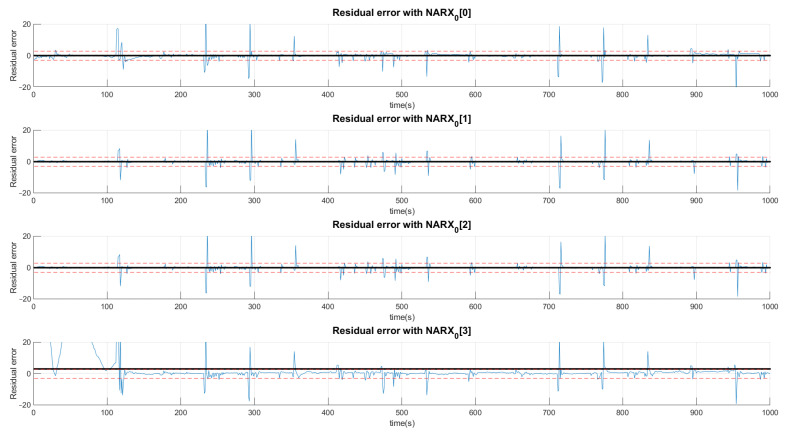
Production of plant residues for the networks expert to detect no presence of faults (NARX${}_{0}$[0]) and for NARX expert to Faults F1, F2 and F3. All of these are produced in the first layer (Layer 0) of the decision tree.

The example shown in Figure 11 indicates the production of residue when the valve system is under the condition of the first fault. The black signals represent the average value of the residue in the presented range and, the dotted lines, the acceptance thresholds for that type of fault. The first graph defines the limits of a signal considered normal for the first layer determined by the average of the signal predicted by the NARX network with half a standard deviation upwards and downwards. The other graphs perform the same procedure with the difference that neural networks are specialists in detecting faults. In this sense, the neural network that offers the least residue, already trained for this purpose, is the first neural network of faults.

The signature is acquired by the standard, in which the mean of the residues are located above +1, below −1 or between the thresholds 0, which depend on the size of the defined deviation.

**Figure 12:**
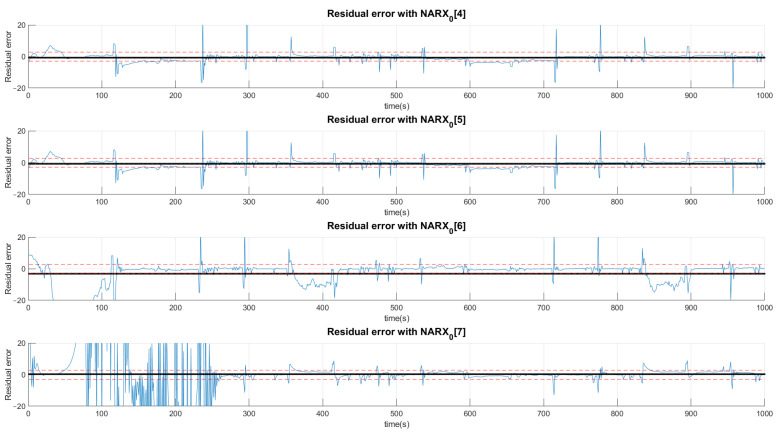
Production of plant residues when NARX networks expert to Faults F4, F5, F6 and F7 are used. All of these are produced in the first layer (Layer 0) of the decision tree.

**Figure 13:**
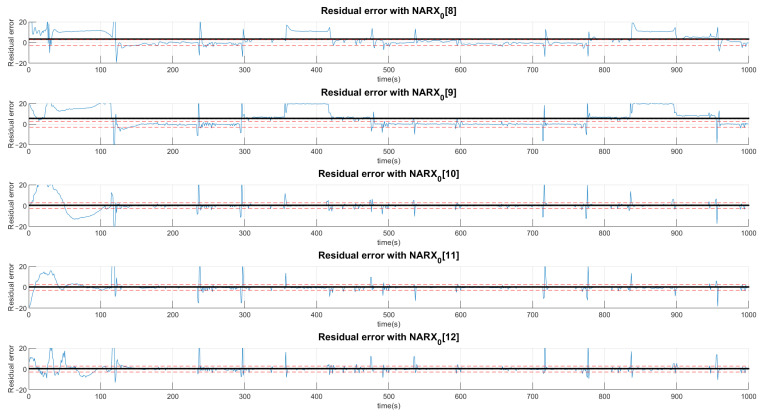
Production of plant residues when NARX networks expert to Faults F8, F9, F10, F11 and F12 are used. All of these are produced in the first layer (Layer 0) of the decision tree.

The choice of high deviations can cause many false negatives since the faults are of low intensity and would not be captured by such a high variation. Thus, we chose to choose values less than a deviation, even if it detects false positives, as they would be confirmed or denied by the various networks. Thus, a signal can be characterized by more than one type of fault or even a normal situation. Concentrating on the example of Fault F1, for the first layer of networks shown in Figure 11, Figure 12 and Figure 13, it is observed that a group of networks indicates what the proper analysis of the fault may be. In this way, the indices presented refer to the order in which each network is able to classify each fault. For example: (NARX${}_{0}\left[1\right]$) represents the Layer 0 (zero) NARX which is the specialist in determining Fault 1. That is, if the data captured is from Fault 1 (Type 1, Intensity 1).

Figure 11, Figure 12 and Figure 13 show the residual values when the same data from the sensors with the same fault are presented to different NARX networks. Each NARX specializes in a different type of fault. Figure 11 shows the residues for specialists in the signal without fault (F0) and those responsible for detecting Faults 1 (F1), 2 (F2) and 3 (F3). Figure 12 for Faults F4, F5, F6 and F7; Figure 13 for F8, F9, F10, F11 and F12. Through the more detailed analysis of the representative graphs in Figure 11, Figure 12 and Figure 13, we can obtain the signatures by positioning the black continuous line, which represents the average of the residue and the dotted lines that determine the variation around the average value. Depending on the location, the signature indicates the activation of each NARX network. If they have values of 0, they will be marked as a possible diagnosis. Those that are different are immediately discarded. All of this methodology is dependent on the viewing window and they need to always choose the same observation interval to make the diagnosis.

**Figure 14:**
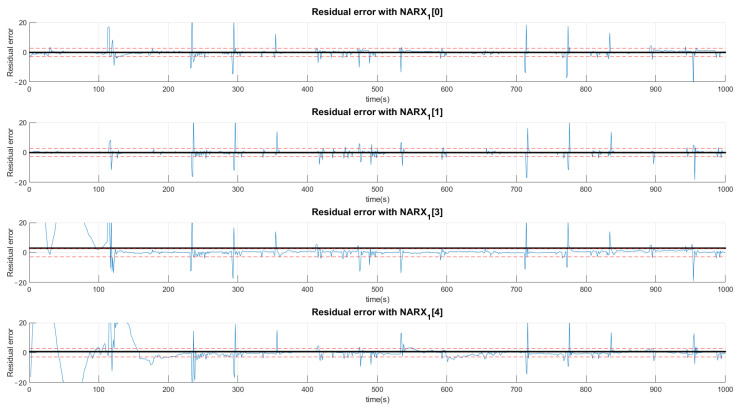
Residual analysis with the second layer of the network excluding those that did not have limits close to zero in the first layer. In this excerpt, residual values are presented for the situations: no faults (NARX${}_{1}$[0]), Faults F1, F3 and F4.

**Figure 15:**
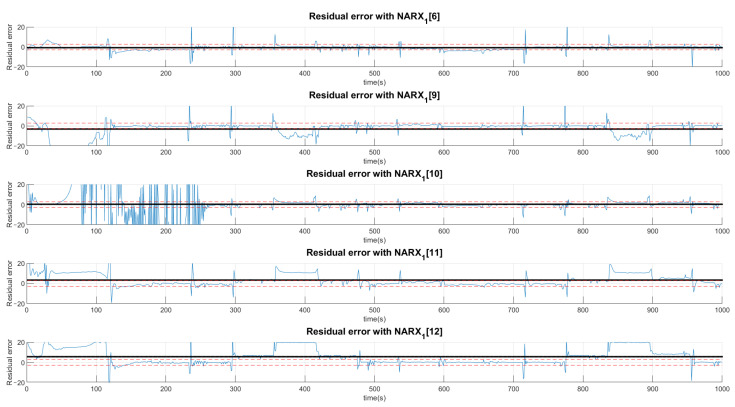
Residual analysis with the second layer of the network excluding those that did not have limits close to zero in the first layer. In this excerpt the residual values for the situations are presented: Faults F6, F9, F10, F11 and F12.

For the second layer of the tree, the analysis is only for residues that had their average values within the limits of acceptance of a possible diagnosis. In this sense, Figure 14 analyzes F0, F1, F3 and F4 and Figure 15 analyzes the possibilities of Faults F6, F9, F10, F11 and F12.

The $G_{1}$ fault set, which contains the faults diagnosed by this pattern is, Equation (1):

(1) $$G_{1}=\left\{0,1,3,4,6,9,10,11,12\right\}$$

What defines the experts for each fault is only the specialized NARX network architecture for each fault. In this situation, the correspondence of the neural network to the first layer can be verified through the first column of the indices shown in Figure 9, with its characteristics shown in Figure 10 and summarized in Table 1. As an example, note that the (NARX${}_{0}$[0]) (specialist in detecting signal without fault) represents the neural network number 45 for the first layer. In turn, network 45 defines an open-loop neural network with six delays at the output and five hidden neurons.

It is not yet possible to adequately isolate the fault using this analysis. Although the F1 Fault belongs to this set, other specialists produce a similar residue that prevents them from being distinguished.

The distinction procedure is continued through a second layer. In this situation, the possibilities of defining other faults that do not belong to $G_{1}$, are eliminated, determining that the subscriptions of the networks belonging to the group with the second best Train Rule (TR02) that were in $G_{1}$ are analyzed on a second level. The results for this second layer are specified in Figure 14 and Figure 15.

With layer analysis, there is no problem with minor or major residual errors. The important thing is that the residues that do not represent the standard are discarded until it is no longer possible to isolate a fault.

**Figure 16:**
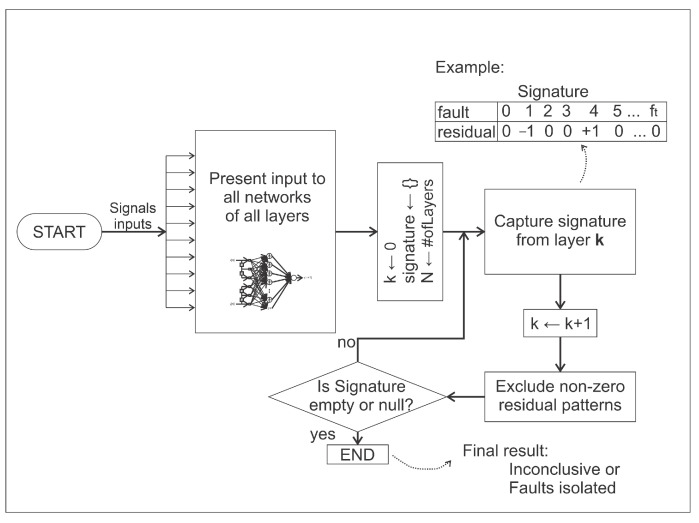
Iterative process of obtaining signatures and fault isolation.

The entire fault isolation process can be described as Figure 16. Initially, the input signals previously obtained from the process are presented to all networks of all layers. The number of layers is a parameter to be chosen. The greater the number of defined layers, the greater the insulating capacity. After this stage, the signature is continuously updated until the moment when it is no longer possible to find a signature with values of −1 or +1 (isolated faults) or all faults situations are discarded (inconclusive).

**Figure 17:**
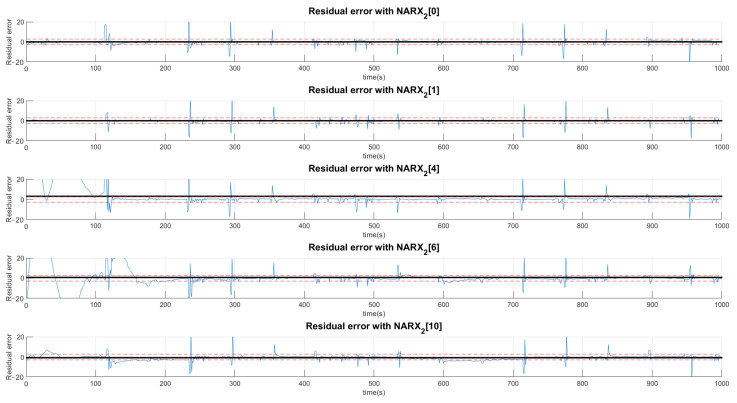
Residual analysis with the third network layer (Layer 2), excluding those that did not have limits close to zero in the second layer.

After performing the analysis for Layer 2, only 5 residue patterns fit the model shown in Figure 17. Fault options are limited to patterns 0 (normal situation), 1 (F1 Fault, with Intensity 1), 4 (F1 Fault, with Intensity 4) and 10 (F3 Fault, with Intensity 2). The number 6 (F2 Fault, with Intensity 2) will be discarded for a new layer since it is not within the limits that classify it.

This methodology presents the steps to be developed in the isolation of faults. In the graphs presented, a signal with the F1 Fault was selected, with a length of 1000 s. In this situation, the intensity of the fault is small enough to be confused with the normal situation itself; however, until the analysis of the third layer, the situation of this fault has not been ruled out.

**Figure 18:**
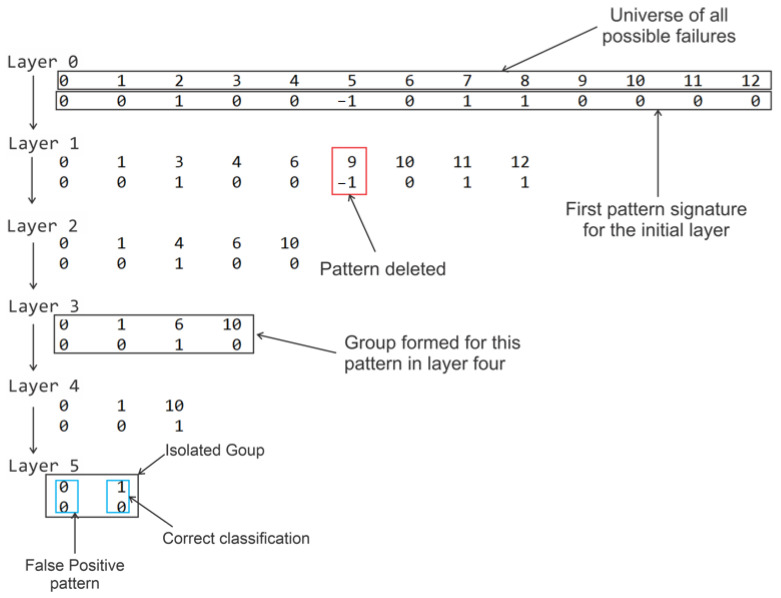
Fault isolation process with the construction of a decision tree for the standard with Fault F1 and Intensity 1.

A complete six-layer analysis of this fault is analyzed for that same interval. The reading of these results can be viewed in the shape of a tree, in which the process of eliminating the patterns is aided by the association of signatures, Figure 18.

In Layer 0 the universe of all situations that may occur from previously emulated faults are present. From this set, below, is the result of the residues of all types represented by a complete signature. It is observed that in 2, 5, 7 and 8, there are behaviors other than zero, which proposes the disposal of these situations. In each layer, the same analysis is performed until there is nothing more to be discarded.

In Layer 5 there is the intended outcome intended in this methodology, which is the greatest possible fault isolation. In the example presented, the fault is difficult to distinguish because it is an example with a low intensity that can be confused both with the normal situation and with all other faults of small intensities in the complete set studied. However, all other situations could be ruled out and, in an intervention to correct the device, the diagnoses considered may be the ones isolated by this methodology.

Regarding the computational effort, the time required to perform this analysis is constant depending on the number of layers, since all data can be presented to all layers in a single step. In addition, when a fault is discarded, the upper layer neural networks that reproduce the residue are turned off and only the cases being evaluated are still under analysis.

## 5. Conclusions

The present work presented a methodology on how to diagnose fault through the isolation using residual patterns and the construction of a decision tree. This proposal takes advantage of the knowledge acquired in a previous analysis of emulated fault patterns so that in an analysis step they can be considered or discarded until the separation of the standards is irreducible. The practical process of selecting specialists with various neural network architectures that could be trained and classified as to the best diagnosis on the behavior of a device in the industrial process, which in this case was a pneumatic valve, was also exposed. Given the above, the methodology proved to be efficient in achieving, in a simplified way, the restriction of the diagnosis of possible faults that may occur. The methodology has as a limitation a previous study, in offline mode, of device tests with the presence of faults. This procedure is not always allowed for control systems as it is necessary for the plant to interrupt current processes. Future work can explore a set of faults with different natures in processes involving other devices and the equipment operating history to make the methodology more comprehensive. In these works, a performance analysis can be verified using different time windows to obtain signatures and confirmation that the fault isolation is still correct.

## Figures and Tables

**Table 1:** Correspondence between the type of failure including the intensity values with the best response for the production of residue.

Fault Type/Intensity	0	1	3	4	5	6	10	11	12
Network	45	48	40	35	17	40	35	36	31
